# Food Deprivation, Body Weight Loss and Anxiety-Related Behavior in Rats

**DOI:** 10.3390/ani6010004

**Published:** 2016-01-07

**Authors:** Silke Dietze, Katarina R. Lees, Heidrun Fink, Jan Brosda, Jörg-Peter Voigt

**Affiliations:** 1 Institute of Pharmacology and Toxicology, School of Veterinary Medicine, Freie Universität Berlin, Koserstr. 20, Berlin 14195, Germany; silke.dietze@fu-berlin.de (S.D.); heidrun.fink@fu-berlin.de (H.F.); 2 School of Veterinary Medicine and Science, University of Nottingham, Sutton Bonington Campus, Loughborough LE12 5RD, UK; katarinalees90@gmail.com (K.R.L.); peter.voigt@nottingham.ac.uk (J.-P.V.)

**Keywords:** body weight, rat, modified open field, elevated plus maze, food deprivation, welfare

## Abstract

**Simple Summary:**

Food deprivation protocols are frequently used in behavioral studies. However, there is limited evidence as to when food deprivation compromises animal welfare. Regarding the refinement of experiments involving animals, this study investigated the effects of food deprivation on body weight loss and behavior in male and female rats. Sex difference in behavior and motivational state after food deprivation is the main finding of the study. The data highlights the need for tailored pilot experiments to evaluate the impact of food deprivation on animals with regard to the 3Rs principles (replacement, reduction, refinement) in animal science.

**Abstract:**

In behavioral studies, food deprivation protocols are routinely used to initiate or maintain motivational states that are required in a particular test situation. However, there is limited evidence as to when food deprivation compromises animal welfare. This study investigated the effects of different lengths of food deprivation periods and restricted (fixed-time) feeding on body weight loss as well as anxiety-related and motivated behavior in 5–6 month old male and female Wistar rats. The observed body weight loss was not influenced by sex and ranged between 4% (16 h deprivation) to approximately 9% (fixed-time feeding). Despite significant body weight loss in all groups, the motivation to eat under the aversive test conditions of the modified open field test increased only after 48 h of food deprivation. Long-lasting effects on anxiety as measured in the elevated plus maze test 24 h after refeeding have not been observed, although fixed-time feeding could possibly lead to a lasting anxiogenic effect in female rats. Overall, female rats showed a more anxiolytic profile in both tests when compared to male rats. Despite these sex differences, results suggest that food deprivation is not always paralleled by an increased motivation to feed in a conflict situation. This is an important finding as it highlights the need for tailored pilot experiments to evaluate the impact of food deprivation protocols on animals in regard to the principles of the 3Rs introduced by Russell and Burch.

## 1. Introduction

Russell and Burch’s 3R principle—Replacement, Reduction and Refinement—has become increasingly important in designing and conducting animal experiments [1,2,3]. Food and water restriction and deprivation procedures are an essential part of many protocols in behavioral neuroscience. These include protocols for appetitive learning, conflict procedures, impulse control, but also the study of feeding behavior itself [4,5,6,7,8,9]. Whereas in behavioral studies food restriction procedures are often being used to change motivational states, toxicity studies or preclinical acute drug screening tests require fasting in order to exclude an effect on diet-dependent substance bioavailability and absorption [10,11,12]. Considering the widespread use of food deprivation procedures in animal research, relatively little systematic studies have been conducted as to what extent these procedures interfere with animal welfare. Fasting in rodents is accompanied with body weight loss, and loss of body weight is regarded as a surrogate parameter for welfare. Food deprivation periods of 24 h and 48 h are the most frequently used protocols [13], and a 48 h fast can lead to up to 20% body weight loss [14]. A 20% loss of body weight is regarded as critical and has been defined as one of the humane endpoints in several international guidelines [15,16,17]. The guidelines set by the European Parliament (Directive 2010/63/EU [18]) state a severity classification of procedures in laboratory animals. The severity of a food deprivation period of 24 h in rats is defined as “mild”, and 48 h of food deprivation is defined as “moderate”. Yet, this grading has not been sufficiently verified by corresponding experimental data. Still, it has to be considered that restriction schedules may improve longevity [19,20]. However, food deprivation or restriction is associated with increasing metabolic and psychological stress, e.g., animals may experience chronic hunger [19,21].

The first aim of this study was to assess the maximum body weight loss in rats following 16, 24 and 48 h fasts. In addition, restricted feeding, *i.e.*, providing either less food or a shorter time when food was available, was also included in this part of the study. Restricted feeding is mostly used in long-term experimental studies, e.g., operant conditioning procedures [22] or in toxicity studies [10]. Generally, a positive correlation between the duration of fasting and the response rate is assumed [23]. To our knowledge, only a few studies have looked into the effects of restricted feeding on species-specific unconditioned behavior [22,24]. Heiderstadt *et al.*, (2000) fed rats at a level to maintain a 20% reduction of their initial body weight and found increased activity in an open field test alongside increased serum corticosterone levels as measured 37 days after the start of the experimental period [22].

The issue of using severe food deprivation in experimental situations, in particular conditioning, has already been discussed in the past [25]. Moran, (1975) brings forward the argument that severe food deprivation could change the motivational state to an extent that it would interfere with species-specific behaviors and thus with multiple variables during conditioning [25]. Such motivational changes could therefore become a confounding variable in behavioral testing, e.g., in protocols of learning and memory testing. This applies in particular to experimental situations where subjects are food deprived and food items are being used as reward.

Therefore, a second aim of the present study was to investigate effects of food motivation in a conflict situation, which does not include conditioning and thus reduces the number of variables. To this end, we used an experimental set up based on the conflict between neophobia/anxiety and hunger [26]. Our aim was to investigate the relationship between the duration of fasting and the motivation of an animal to overcome a potentially aversive situation for the benefit of getting food. For balancing the anxiety of the rat against the motivation of hunger a modified open field (mOF) test [27], also known as novelty induced hypophagia (NIH) test [28,29], was used. The mOF was extensively validated in our laboratory and represents a conflict model, which is a valid measure of anxiety-related behavior as anxiolytic drugs stimulate food intake in such an aversive situation [27].

To investigate possible long lasting effects on emotional states of the animals, we exposed rats to the elevated plus maze (EPM) test of anxiety-related behavior. The EPM test is a subtle and commonly used test to assess anxiety-related and exploratory behavior in rodents [30]. The test has been extensively validated and evaluated [31,32,33,34]. It has been demonstrated that preceding stress has a significant impact on measures of anxiety-related behavior in this test [35,36,37], which makes it a robust animal model of state anxiety [36,38].

We hypothesize that food deprivation will lead to a significant reduction in body weight, accompanied by reduced anxiety-related behavior in the mOF test, *i.e.*, an increased motivation to feed in an aversive environment. The magnitude of behavioral effects should correlate with the duration of food deprivation.

The results of the study on hand may help to gain new insights for the refinement of various feeding protocols in laboratory animals, which will consequently reduce stress in the animals and enhance their wellbeing during housing and in the experimental setting.

## 2. Experimental Section

### 2.1. Animals

The experiments were performed in accordance with the guidelines of the German Animal Protection Law and had been approved by the Berlin State Authority (“Landesamt für Gesundheit und Soziales” (LaGeSo); G 0153/12). The ethical acceptability of the animal experiments is a crucial part of the application, which was discussed with the Animal Welfare Officer of the Freie Universität Berlin and subsequently reviewed by an ethical commission assisting the LaGeSo Berlin. The potential of implementing the concept of the 3Rs was rigorously researched during each part of the present study. All experimenters involved were well trained and experienced in the use of laboratory animals and their behavior.

A total of 51 naive male (*n* = 25; Ø 440 g ± 30 g) and female (*n* = 26; Ø 260 g ± 16 g) Wistar rats (Harlan Laboratories, Horst, the Netherlands) aged 5–6 months were used. Prior to the experiments, the animals were group-housed (*n* = 2–4) in type IV Macrolon cages (59 × 38 × 20 cm, floor area 1.815 cm^2^; Ehret, Wandlitz, Germany) with dust-free hardwood bedding (Hygienic Animal Bedding, J. Rettenmaier & Söhne GmbH und Co. KG, Rosenberg, Germany) under standard conditions (room temperature 22 ± 2 °C; relative humidity 55% ± 10%) on a 12 h light/dark schedule (lights on at 6:00 am). Home cages were enriched by paper tissues and metal tubes as hiding places. Before and during experiments the rats received the standard lab chow to which they were accustomed (V153x R/MH:19% protein, 3.3% fat, 4.9% fiber, 6.4% ash, trace elements, and vitamins; ssniff, Soest, Germany) and tap water, and were handled and weighed every other day. Once weekly, the home cages were cleaned and equipped with new bedding by a professional animal keeper. Animals were free of pathogens according to Federation for Laboratory Animal Science (FELASA) recommendations and their health status was monitored quarterly.

### 2.2. Experimental Design

#### 2.2.1. Food Deprivation and Fixed-Time Feeding Schedule

After an initial body weight measurement, which allowed us to exclude any significant outliers, the animals were randomly allocated to the five independent experimental groups of 16 h, 24 h, 48 h of food deprivation (no access to food), 4 days of food restriction (fixed-time feeding; FF), or *ad libitum* feeding (control group). Sixteen hours before starting the experiments, the rats were transferred to single-housing (Macrolon cages type III; habituation period) enriched by metal tubes as hiding places. In the fixed-time feeding group, animals received food for 4 h daily (8:00–12:00 am) for four consecutive days [27]. The *ad libitum* fed control group underwent the same handling procedures (e.g., single housing, body weight measurement). Water was freely available at all times. With the exception of the control group (five males, six females), each group consisted of five males and five females. Group sizes were calculated by G*Power 3 (Heinrich-Heine-Universität Düsseldorf, Düsseldorf, Germany) [39].

#### 2.2.2. Body Weight Measures

Body weight in relation to mOF testing was measured immediately before the food deprivation commenced and following the 5 min exposure to the test, *i.e.*, before refeeding. Body weight before fasting or fixed-time feeding was set at 100% for each animal and their body weight at the time of mOF was then compared to this.

#### 2.2.3. Behavioral Experiments

All experiments were conducted in a sound-attenuated room between 8:30–12:30 am. Animals were habituated to the room for 1 h before starting the respective experiment. The behavior of each rat was recorded and analyzed using a computer-based tracking system (VideoMot 2; TSE-Systems, Bad Homburg, Germany). Testing arenas were cleaned with Meliseptol^®^ between each animal. The timing of food deprivation periods was staggered, to ensure that animals of the individual experimental groups had fasted for the same time at the beginning of the behavioral testing, which was performed during the last hour of food deprivation.

#### 2.2.4. Modified Open Field (mOF) Test

The mOF test was performed as described by Rex *et al.*, (1998) [8]. Briefly, the rats were individually placed in one corner of a brightly illuminated (350 lux) white open field (100 × 100 × 40 cm) facing the center of the open field, in which familiar food pellets (standard chow) were available. The rats were observed for 5 min and their behavior was analyzed for the following parameters: incidence of food intake (% of rats feeding per group), latency to initial food contact (s), distance traveled (cm, horizontal locomotion), and number of rearings (vertical activity). At the end of each experiment, animals were returned to their home cages and to *ad libitum* feeding.

#### 2.2.5. Elevated Plus Maze (EPM) Test

Twenty-four hours after completion of the mOF experiment and refeeding, the animals were tested for anxiety-related behavior using an EPM. The maze consisted of two open arms (50 × 15 cm) and two enclosed arms (50 × 15 × 10 cm), which were connected by a central platform (15 × 15 cm; height of the maze: 64 cm). Illumination was 250 lux on the surface of the open arms, 60 lux in the closed arms and 200 lux on the central platform. The rats were placed on the central platform facing the corner between open and closed arm, allowing equal opportunity of entering one of these arms. Each animal was observed for 5 min. As classical parameters related to anxiety behavior, the number of entries into the open arms in relation to total entries, and the time spent in the open arms (s) were recorded. In addition, the total distance traveled (cm) and number of rearings were measured (these parameters are not independent from anxiety in the EPM). The number of head dips was monitored as a parameter of risk assessment.

#### 2.2.6. Data Presentation and Analysis

Data was analyzed and presented using SigmaPlot 11 software (Systat Software, Erkrath, Germany). Body weight data was analyzed by Student’s paired t-tests. A Fisher’s exact test was used to evaluate the incidence of food intake in the mOF and an alpha adjustment was applied for multiple comparisons to the control group. All other behavioral data (mOF and EPM) was analyzed by two-way ANOVA (sex, feeding condition) followed by *post hoc* analysis using the Holm-Sidak method. *p* < 0.05 was considered statistically significant. Data is presented as mean values plus standard error of the means (S.E.M.).

## 3. Results

### 3.1. Effect of Food Deprivation and Fixed-Time Feeding Schedule on Body Weight Change

**Figure 1:**
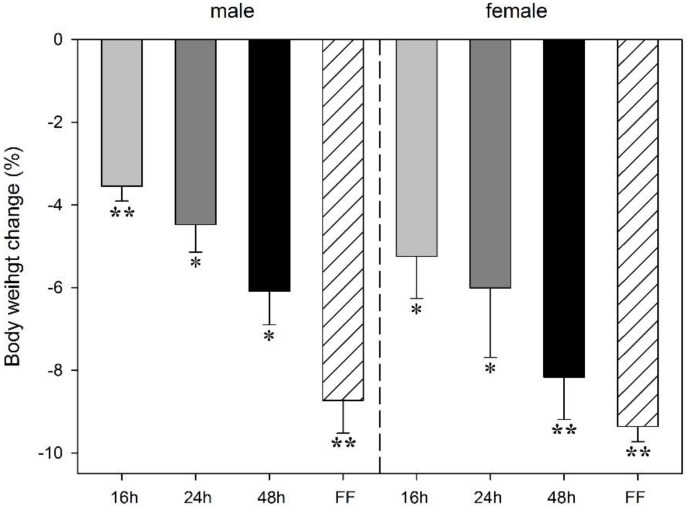
Effect of food deprivation (16 h, 24 h, 48 h) or fixed-time feeding schedule (FF; 4 h for 4 days) on body weight change in male and female rats. Expressed are the mean body weight changes (%) in relation to animal body weights at the onset of deprivation period or food restriction. Data is mean -S.E.M. * *p* < 0.05; ** *p* < 0.001 before *vs.* after food deprivation.

Food deprivation led to a decrease in body weight in both male and female rats. The animals’ weight loss increased with the duration of fasting and was greatest under fixed-time feeding conditions. The effects varied between a 3.6% (*p* < 0.001, males) and 5.3% (*p* < 0.004, females) after 16 h of deprivation up to a 6.1% reduction (*p* < 0.003 males) or 8.2% (*p* < 0.001, females) after 48 h. The most pronounced weight loss occurred after the fixed-time feeding schedule. Four daily feeding periods lasting 4 h each induced body weight losses of 8.7% in male and 9.4% in female rats (*p* < 0.001; Figure 1). Two-way ANOVA did not reveal any sex effect on body weight change during food deprivation. There were negligible body weight changes in the control groups during the experimental period (+0.46% in males, +0.73% in females).

### 3.2. Effect of Food Deprivation and Fixed-Time Feeding Schedule on the Performance in the mOF Test

**Figure 2:**
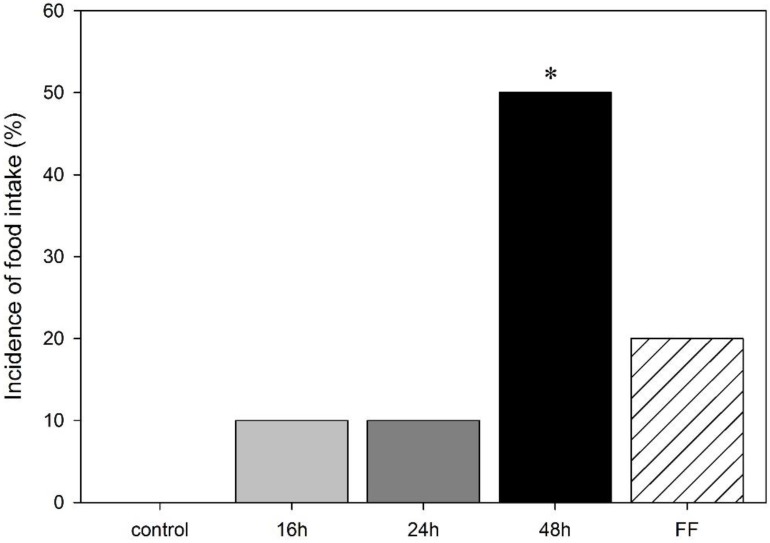
Percentage of rats feeding in the modified open field test in male and female rats following food deprivation (16 h, 24 h, 48 h), fixed-time feeding schedule (FF; 4 h for 4 days) or free feeding (control). Data is mean, * *p* < 0.05 *vs.* control.

As the impact of deprivation on body weight was not sex-specific, the incidence of food intake in the mOF was analyzed for males and females together. While 16 h and 24 h fasts (one out of 10 animals (10%) each) as well as a fixed-time feeding schedule (2/10 (20%)) showed no effect on the rats’ incidence to eat when compared to controls (0/11 (0%)), a 48 h fast significantly increased the number of rats feeding in the arena to (5/10 (50%)); as compared to controls (*p* < 0.05; Figure 2). No differences in the latency to start feeding were observed.

**Figure 3:**
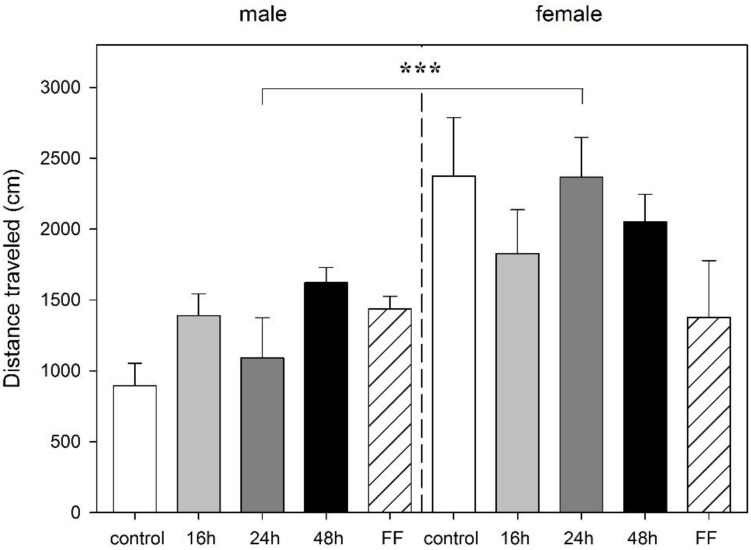
Effect of food deprivation (16 h, 24 h, 48 h), fixed-time feeding schedule (FF; 4 h for 4 days) or free-feeding (control) on spontaneous activity in the modified open field test in male and female rats. Data is mean +S.E.M. Two-way ANOVA showed sex significance, *** *p* < 0.001, but no food deprivation significance.

In male or female rats, the length of food deprivation had no effect on the distance traveled. However, an interaction between sex and deprivation period (F_4,40_ = 2.89, *p* = 0.035) and a significant main effect of sex (F_1,40_ = 17.65, *p* < 0.001) were found with females rats traveling a longer distance in the mOF (Figure 3). Food deprivation had no effect on rearings. One female rat of the FF group was excluded from the analysis because of malfunction of the tracking software.

### 3.3. Effect of Food Deprivation and Fixed-Time Feeding Schedule on the Performance in the EPM Test

**Figure 4:**
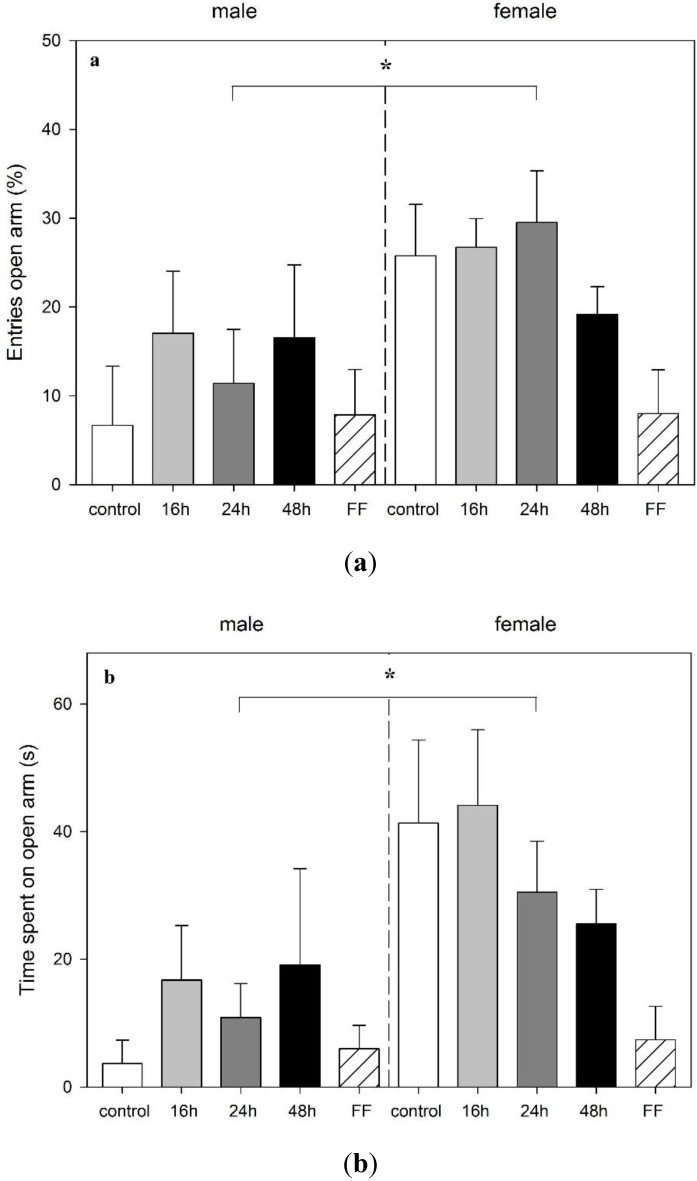

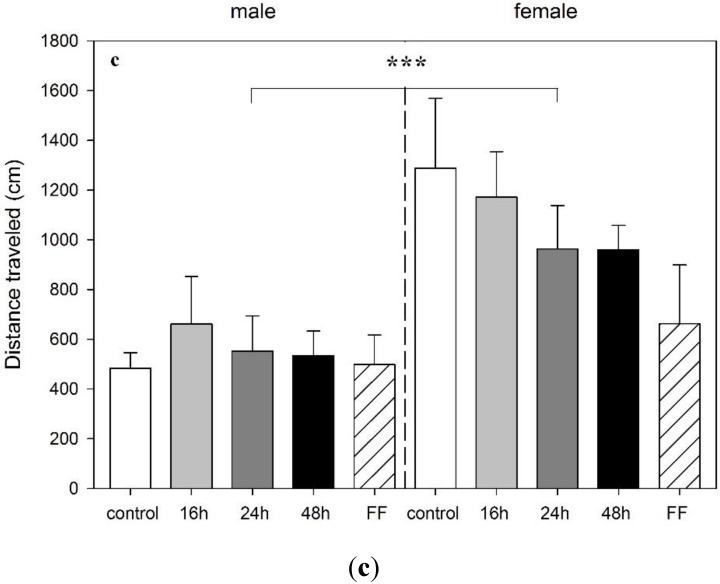
Effect of food deprivation (16 h, 24 h, 48 h), fixed-time feeding schedule (FF; 4 h for 4 days) or free-feeding (control) on the performance in the elevated plus maze test in male and female rats. (**a**) Percentage of open arm entries; (**b**) time in open arms; and (**c**) distance traveled are shown. Data is mean +S.E.M. Two-way ANOVA showed sex significance, (A and B: * *p* < 0.05; C: *** *p* < 0.001) but no food deprivation significance.

Twenty-four hours following mOF exposure and returning to *ad libitum* feeding, all rats were tested in the EPM. None of the preceding food deprivation schedules had an impact on anxiety-related behavior in the EPM when rats were exposed to the test following the refeeding period (Figure 4). However, a significant main effect of sex on anxiety-related parameter entries and time spent in open arms (entries (%): F_1,41_ = 7.32, *p* = 0.01; time (s): F_1,41_ = 10.23, *p* = 0.003) was found. Females showed an increase in these parameters, *i.e.*, they visited the aversive arms more frequently, suggesting anxiolytic behavior (Figure 4a,b). Although there was no overall effect of feeding on the EPM behavior, female rats, previously exposed to fixed-time feeding, showed a tendency to significance for spending less time on the aversive open arms (*p* = 0.083) (Figure 4b).

As in the mOF, females traveled a longer distance in the EPM when compared to males (F_1,41_ = 16.89, *p* < 0.001) (Figure 4c). Food deprivation had no effect on rearings or head dips in this test.

## 4. Discussion

In this study, body weight changes resulting from food deprivation and food restriction and subsequent behavioral changes have been assessed in rats. There are currently a limited number of studies analyzing these parameters together, and therefore the present study aims at providing a more comprehensive view on the effects of food deprivation and food restriction in the rat. The main finding is that food deprivation and a fixed-time feeding caused a significant and sex-independent loss in body weight, but only a 48 h food deprivation had a measurable effect during behavioral testing. This finding was not expected because deprivation periods in the range of 16 h and 48 h are widely used in behavioral studies [13].

The average 8% weight loss (the observed maximum was 13% in a single rat) following the 48 h deprivation period as reported here is in keeping with previous reports [40,41,42]. However, others have reported a higher average weight loss of 15% in Wistar rats following only 24 h of deprivation [43]. In other rat strains, even higher reductions up to 18%, have been reported following a 24 h fast [44,45,46,47]. Thus, strain and substrains that may exist in various laboratories could be a variable that contributes to the extent of body weight loss following food deprivation. Regarding the moderate weight loss seen in the present study, compensatory water consumption can be excluded, as various studies have shown that body weight decrease during deprivation periods of 3 and 4 days is paralleled by a decreased water intake [41,48]. Another variable could be the age of the animal, with younger rats losing a higher percentage of body weight than older rats. The evidence for this comes from a study by Li and Wassner [49], where younger rats lost 29% body weight following 24 h of food deprivation.

Therefore, we suggest performing pilot studies to ensure that weight loss does not exceed the permitted weight loss limit imposed by the regulatory body and/or animal ethics committee to prevent undue suffering to the animals.

A strong effect on body weight could also be indicative of a severely unbalanced motivational state, which could add a stochastic element to behavioral testing and compromise the interpretation of behavioral data [25]. This aspect was approached with the behavioral part of the present study, where we tried to establish the length of a deprivation period that would be required to increase the motivation of a rat in a conflict situation and whether a mild fixed-time feeding regimen would also be suitable to generate this motivation. Therefore, the mOF test was used in the present study. This test is based on the interplay between the rats’ motivation to feed, as manipulated by food deprivation or restriction, and anxiety, induced by exposure the aversive open field [27]. By increasing the time of food deprivation, the test allows us to identify the threshold where the motivation to feed overrides the anxiety-induced protective behavior as expressed by the avoidance of the center of the arena where the food has been placed. Surprisingly, this threshold was not reached with fasts shorter than 48 h where 50% of the rats went to the center and started to eat. Based on previous results [27], we expected to find this threshold at a rather shorter deprivation period. Various factors could account for the discrepancy of these studies, including the averseness to the test arena, the novelty of the presented food items, age of the animals, or the substrain of rats that were used. As discussed before, age could contribute to the relatively small weight loss following food deprivation and hence a reduced motivation to feed. The averseness to the present test conditions was not different compared to Rex *et al.*, (1998), and the presented chow was familiar to the rats, whereas in other versions of this test novel food had been presented [50,51]. Previous studies in our lab demonstrated that between 10% and 100% of rats from various strains started feeding following 20 h of deprivation [27,52,53]. Moreover, substrains of Wistar rats differ considerably in their food intake after a 20 h deprivation [53].

The present study suggests that despite a significant loss of body weight, the motivation to feed under anxiogenic conditions requires a certain level of body weight loss. A discussion of our findings in the context of previous studies, both from our lab and from other groups, revealed that this level seems to depend on a variety of factors. Hence, customized pilot studies are recommended assessing which level of body weight loss actually impacts on the motivational state in a given experimental situation. This could also minimize detrimental interferences due to “overdeprivation” with motivational states as for example during conditioning experiments. The outcome of these pilot studies could actually improve the welfare of the rats by the avoidance of unnecessarily long fasting periods.

In the fixed-time feeding group, rats lost more body weight than the 48 h fasted rats. However, in contrast to food deprivation, four days of fixed-time feeding did not affect feeding in the mOF. Although in the current study the weight loss was the highest in this group, it was still lower compared to studies where behavioral effects have been reported [54,55]. The lack of correlation between weight loss and behavior in the mOF indicates that weight loss *per se* is not a good indicator of the motivational and/or emotional state of the rat. Unlike our results, Inoue *et al.*, (2004) could demonstrate a reduction of anxiety tested on the EPM in Wistar rats after restricted feeding [54]. However, with only 2 h daily feeding time, their food restriction regime was more stringent than ours. Similarly, food restriction for 7 days maintaining 85% of the body weight resulted in an anxiolytic effect in male Lister Hooded rats [55]. In another study, male rats maintained at 80% of their initial body weight made more entries into the aversive inner zone of an open field, which could be interpreted as an anxiolytic effect. Only repeated exposure to the test arena revealed increased locomotor activity in these rats [22]. However, in the light of increased serum corticosterone levels in these rats, the interpretation of the behavioral data is not straightforward, because high levels of stress hormones are usually associated with increased anxiety [56]. Fixed-time feeding initiates a cascade of neurobiological and metabolic events, which can prevent obesity in rodents [57]. Restricted feeding, in particular fixed-time feeding, has multiple effects on a variety of physiological and behavioral systems which include the entrainment of circadian rhythms and adjustments in plasma corticosterone levels in anticipation to feeding [58,59]. Fixed-time feeding causes adaptations in the CNS that include circadian oscillators [60], but these adaptations are not confined to one brain structure and are more evident in the thalamus, hippocampus and hypothalamus [61]. One would expect that these neural adaptations, although likely to emerge early on in rats, would develop over time. Hence a gradual initiation of the procedure (“training”) could possibly have a stress-reducing effect. However, revealing time pattern and plasticity of fixed-time feeding-induced neural and behavioral changes requires further investigation.

The third part of our study was aimed to answer the question whether in refed rats the experience of a previous period of food deprivation or restriction could lead to lasting behavioral consequences. Physiological changes occur during and after a food deprivation period, especially during the refeeding period [41]. Moreover, food deprivation is a potential stressful event. Thus, acute deprivation for 24 h, but not for 6 and 16 h, has been reported to induce an elevation of plasma corticosterone levels in Sprague Dawley rats [62]. Not only acute food deprivation but also chronic restriction have an impact on several plasma hormone levels, with an increase of corticosterone in Wistar rats and in other rat strains [19,63], although contrasting findings suggests that a significant corticosterone increase in Wistar rats does not occur before at least 5 days of food deprivation [64,65].

In our study, only the female fixed-time fed group showed possibly lasting behavioral effects following refeeding as expressed by a tendency of anxiety related behavior in the EPM. Such an effect, if true, would contradict previous findings where exposure of female Sprague-Dawley rats to cycles of caloric restriction followed by refeeding did not influence anxiety related behavior [66]. However, the present study cannot rule out any long lasting behavioral effects, in particular in behaviors not directly related to anxiety, as those have not been investigated here.

The observed overall anxiolytic behavior in females on the EPM is in keeping with previous studies [67,68,69]. Despite the sex effect on locomotor activity, which is possibly not independent from anxiolysis at least in the EPM [32], food deprivation and restriction *per se* had no impact on the locomotor activity in the respective test groups. This coincides with earlier findings [70], demonstrating in Sherman rats that locomotor activity was not affected by food deprivation whereas other forms of exploratory behavior increased. Similarly in Long-Evans rats, food deprivation did not affect locomotor activity [71].

In further studies on the subject, systematic alteration of experimental variables, e.g., various degrees of deprivation *vs.* various degrees of averseness should be performed. Concomitant measures of stress hormones, but also analysis of home cage behavior might help to further substantiate the effect of various feeding and deprivation schedules on welfare.

## 5. Conclusions

In conclusion, our results could help to improve animal welfare by the refinement of study designs, which involve food restriction or deprivation of experimental animals. The data suggests that weight loss and motivation to feed are not strongly correlated. Our results are in favor of pilot studies to ensure good welfare during the actual behavioral experiments.
